# Hospital Diabetes Meeting 2020

**DOI:** 10.1177/1932296820939626

**Published:** 2020-08-12

**Authors:** Guillermo Umpierrez, Robert Rushakoff, Jane Jeffrie Seley, Jennifer Y. Zhang, Trisha Shang, Julia Han, Elias K. Spanakis, Sara Alexanian, Andjela Drincic, Kristen Kulasa, Carlos E. Mendez, Damon Tanton, Amisha Wallia, Mihail Zilbermint, David C. Klonoff

**Affiliations:** 1 Emory University, Atlanta, GA, USA; 2 UCSF, San Francisco, CA, USA; 3 Weill Cornell Medicine, NY, USA; 4 Diabetes Technology Society, Burlingame, CA, USA; 5 University of Maryland School of Medicine, Baltimore, MD, USA; 6 Division of Endocrinology, Baltimore Veterans Affairs Medical Center, Baltimore, MD, USA; 7 Boston University School of Medicine, MA, USA; 8 University of Nebraska Medical Center, Omaha, NE, USA; 9 UCSD, La Jolla, CA, USA; 10 Medical College of Wisconsin, Milwaukee, WI, USA; 11 AdventHealth Diabetes Institute, Orlando, FL, USA; 12 Northwestern University, Chicago, IL, USA; 13 Division of Endocrinology, Diabetes, and Metabolism, Johns Hopkins University, Baltimore, MD, USA; 14 Johns Hopkins Community Physicians at Suburban Hospital, Bethesda, MD, USA; 15 Johns Hopkins Carey Business School, Baltimore, MD, USA; 16 Mills-Peninsula Medical Center, San Mateo, CA, USA

**Keywords:** diabetes, digital health, glucose, hospital, insulin

## Abstract

Patients with diabetes may experience adverse outcomes related to their glycemic control when hospitalized. Continuous glucose monitoring systems, insulin-dosing software, enhancements to the electronic health record, and other medical technologies are now available to improve hospital care. Because of these developments, new approaches are needed to incorporate evolving treatments into routine care. With the goal of educating healthcare professionals on the most recent practices and research for managing diabetes in the hospital, Diabetes Technology Society hosted the Virtual Hospital Diabetes Meeting on April 24-25, 2020. Because of the coronavirus disease 2019 (COVID-19) pandemic, the meeting was restructured to be held virtually during the national lockdown to ensure the safety of the participants and allow them to remain at their posts treating COVID-19 patients. The meeting focused on (1) inpatient management and perioperative care, (2) diabetic ketoacidosis and hyperglycemic hyperosmolar state, (3) computer-guided insulin dosing, (4) **Coronavirus Disease 2019** and diabetes, (5) technology, (6) hypoglycemia, (7) data and cybersecurity, (8) special situations, (9) glucometrics and insulinometrics, and (10) quality and safety. This meeting report contains summaries of each of the ten sessions. A virtual poster session will be presented within two months of the meeting.

## Introduction

More than 25% of hospitalized patients have a history of diabetes and are at high risk for hospital-related complications, such as increased 30-day readmission rate and mortality.^1-5^ Overall, the cost of hospitalizations among patients with diabetes is more than $123 billion per year in the United States.^6^ As a result, there is a growing interest from healthcare professionals about the inpatient management of patients with diabetes. On April 24-25, 2020, Diabetes Technology Society convened a meeting of 27 expert panelists in diabetes and endocrinology with a goal of discussing the current status and the future directions of diabetes care in the hospital. The meeting’s cochairs were Guillermo Umpierrez, MD, CDE, Robert Rushakoff, MD, and Jane Jeffrie Seley, DNP, MPH, MSN, GNP, RN, BC-ADM, CDCES, CDTC, FADCES, FAAN. Because of the coronavirus disease 2019 (COVID-19) pandemic situation, the meeting was held virtually and one of the sessions addressed timely issues related to the management of hospitalized patients with diabetes and COVID-19 infection.

## Session 1: Inpatient Management and Perioperative Care

Moderator: Guillermo Umpierrez, MD, CDE

Emory University, Atlanta, GA, USA

### Insulin Use in the Hospital (Insulin Dosing)

Amisha Wallia, MD, MS

Northwestern University Feinberg School of Medicine, Chicago, IL, USA

#### Key ideas

- Insulin is the most effective and most well-studied medication for the treatment of diabetes and hyperglycemia in the inpatient setting.
- Although it may cause hypoglycemia and may require increased nursing time and frequent dose adjustments from users who are trained in this type of management, the benefits are well worth its use.
- Better control has been noted in both medical and surgical patients with diabetes and/or hyperglycemia utilizing a weight-based basal, bolus, and supplemental regimen.

#### Summary

Insulin therapy is the recommended approach for the management of hospitalized patients with diabetes or hyperglycemia. Subcutaneous insulin administration following a basal-bolus or a basal-plus regimen is the favored strategy for noncritically ill hospitalized patients, while the use of paper or computerized intravenous infusion protocols is endorsed for those critically ill.^7^ Nonetheless, insulin therapy is known to increase the risk for hypoglycemia and is also often considered cumbersome and complicated, requiring frequent point of care blood glucose (BG) monitoring and intensive staff training and exposure.^8^ Despite the lack of recommendations, the use of oral antidiabetic agents is a common practice in the hospital setting. Although likely effective in some patients with mild hyperglycemia, agents such as metformin and sulfonylureas should generally be avoided in acutely ill patients with contraindications or at risk for lactic acidosis or hypoglycemia.^8^

### Use of Oral Antidiabetic Agents in the Hospital in Patients With Type 2 Diabetes

Guillermo Umpierrez, MD, CDE

Emory University, Atlanta, GA, USA

#### Key ideas

- Insulin is the preferred agent for hospital use in the management of patients with type 1 diabetes (T1D) and type 2 diabetes (T2D).
- The use of dipeptidyl peptidase-4 (DPP-4) inhibitors in medicine and surgery patients with mild-moderate hyperglycemia is safe and effective.
- Randomized controlled trials are needed to assess the inpatient efficacy and safety of other oral and injectable antidiabetics.

#### Summary

Although insulin is still regarded as the preferred method of glycemic control for hospitalized patients with T1D and T2D, several recent clinical studies have evaluated the safety and efficacy of the use of DPP-4 inhibitors alone or in combination with basal insulin in noncritically ill patients with lower insulin requirements (<0.6 U/(kg⊕day)) and mild to moderate hyperglycemia (<200 mg/dL). Results have consistently showed similar levels of glycemic control, compared to basal-bolus insulin regimen, but with fewer hypoglycemic events and a lower number of daily insulin injections.^9-11^ Poor glycemic control has been associated with an increased risk of perioperative complications.

### Outpatient Preparation for Surgery (Outpatient Programs, Dose Adjustments)

Carlos E. Mendez, MD, FACP

Medical College of Wisconsin, Milwaukee, USA

#### Key ideas

- Although glycated hemoglobin (HbA1c) is frequently used as the measure for glycemic control before surgery, correlation between A1c levels and perioperative outcomes has not been consistent. New surrogates for glycemic control, such as fructosamine, show promise to better inform on glycemic control in the short term prior to surgery.
- Processes designed to optimize patients with diabetes at high risk of complications before surgery have shown to improve glycemic control and possibly improve outcomes.
- In terms of medication adjustments before surgery, the U.S. Food and Drug Administration (FDA) now advises that sodium-glucose cotransporter-2 (SGLT-2) inhibitors should be stopped at least three days before procedures.

#### Summary

Although HbA1c has been traditionally used to evaluate the level of control before surgery, new evidence suggests that short-term markers, such as fructosamine, may be better predictors of adverse outcomes after surgery.^12^ Diabetes optimization programs, working in parallel or embedded within preoperative clinics, have shown to significantly improve glycemic control before surgery and reduce complications.^13,14^ Because of the risk of euglycemic diabetic ketoacidosis with the use of SGLT-2 inhibitors around surgery, the FDA recently released a labeling change, advising that these drugs should be stopped at least three days before procedures.^15^ Additionally, DPP-4 inhibitors and GLP-1 agonists may have a more important role in the future perioperative management of diabetes and hyperglycemia.

### Intraoperative/ Post-Anesthesia Care Unit Management

Jeffrey Joseph, DO

Thomas Jefferson University, Philadelphia, PA, USA

#### Key ideas

- In the intraoperative period, the goal is to maintain the BG concentration in the desired target range for that individual (for example, 90-140 mg/dL) while minimizing the degree and duration of hyperglycemia, hypoglycemia, and glycemic variability.
- Clinicians should adjust the target BG range according to the specific physiology of each individual patient. Additional prospective randomized clinical trials are required to determine which patient populations may benefit from specific glucose targets.
- The color and quantity of urine output commonly used to inform renal perfusion or hemodynamic status may not be a reliable indicator in patients experiencing hyperglycemia.

#### Summary

Adequate intraoperative glycemic control has been shown to reduce complications. Ideally, glucose concentrations should be maintained within an individualized set range, while minimizing the degree and duration of hyperglycemia, hypoglycemia, and glycemic variability.^16^ Anesthesiologists and surgeons identify and quantify patient risk factors prior to surgery to minimize the risk of perioperative complications. Although most recommended targets vary in between 90 and 180 mg/dL,^12^ additional prospective randomized clinical trials are required to determine which patient populations may benefit from specific glucose targets. A higher than normal BG target may be appropriate for patients with diabetes experiencing decreased local blood flow due to an ischemic stroke, hemorrhagic stroke, or myocardial infarction, whereas a near-normal BG target may be appropriate in patients undergoing cardiac surgery with satisfactory preoperative BG control. In addition, urine output may be an unreliable measure of renal perfusion during the perioperative period. In patients with significant hyperglycemia, increased urine output from glycosuria can lead to hypovolemia and acute kidney injury or renal failure in the postoperative period.^17^

## Session 2: Diabetic Ketoacidosis and Hyperglycemic Hyperosmolar State

Moderator: Robert Rushakoff, MD

USCF, San Francisco, CA, USA

### History of Diabetic Ketoacidosis and Hyperglycemic Hyperosmolar State

Robert Rushakoff, MD

USCF, San Francisco, CA, USA

#### Key ideas

- The Bradshawe Lecture on Diabetic Coma by Julius Dreschfeld classified the cases of diabetic coma, according to the symptoms, into three groups: drowsiness, alcohol intoxication, and dyspnea.
- The use of insulin in the treatment of diabetic coma at home and in the hospital began around 1945.
- Further treatment methods such as bicarbonate therapy for diabetic ketoacidosis (DKA) continued to be developed.

#### Summary

A foundational reference for the clinical presentation of hyperglycemic crisis is the 1886 Bradshawe Lecture by Julius Dreschfeld. He describes three presentations of “diabetic coma.” The most common one demonstrates dyspnea, severe abdominal pain, and includes Adolph Kussmaul’s description of breathlessness and “expelled air with a chloroform-like smell.” Another occurs in “stout” patients beyond age 40, with a rapid pulse and urine with high sugar but no acetone. These represent DKA and hyperglycemic hyperosmolar state (HHS); the third form described is less clear. Identified precipitants included excessive exercise, physiologic or mental stress, and an exclusively meat diet, with treatment by avoiding the same. In the 1970s, low dose insulin (on average 46 units of insulin) vs high dose (236 units of insulin) was found to cause less hypokalemia and hypoglycemia and became standard. Further studies showed all insulin delivery routes effectively treated DKA, but no improvement with the use of bicarbonate.

### Diabetic Ketoacidosis and Hyperglycemic Hyperosmolar State Management and Treatment Protocols in the Emergency Department/Floor/Intensive Care Unit

Nadine E. Palermo, DO

Brigham and Women’s Hospital/Harvard Medical School, Boston, MA, USA

#### Key ideas

- Several studies have demonstrated that the use of subcutaneous rapid acting insulin given every one to two hours can be effective in the treatment of mild/moderate uncomplicated DKA.
- Classification of DKA severity can be useful in triage as patients presenting with mild uncomplicated DKA may be treated in an intermediate unit as an alternative to the intensive care unit (ICU).
- Special considerations for alternative strategies in the management of hyperglycemic crises may be especially useful during a pandemic, including subcutaneous dosing and extended intervals for glucose monitoring and insulin administration.

#### Summary

The classification of patients by type of hyperglycemic crisis (mild-severe DKA, HHS, or both) determines appropriate therapy with intravenous or subcutaneous insulin and triage location (such as the ICU). Patients in the emergency department (ED) with hyperglycemia but without hyperglycemic crisis should be considered for rapid follow-up programs if available to avoid unnecessary hospitalizations. Fluids, insulin, and electrolyte repletion remain the mainstays of therapy. Patients may benefit from early administration of basal insulin to decrease rebound hyperglycemia after insulin infusions are discontinued.^18^ Studies have shown protocols utilizing subcutaneous insulin every one or two hours are as effective as infusions in patients with mild-moderate DKA.^19^ For safe discharge, patients must have had adequate education, prescriptions and needed supplies, and follow-up scheduled. For patients with COVID-19, in order to conserve personal protective equipment, it is possible to effectively treat mild-moderate DKA with subcutaneous insulin every four hours.

### Pitfalls in the Management of Diabetic Ketoacidosis and Hyperglycemic Hyperosmolar State

Mary Korytkowski, MD

University of Pittsburgh, Pittsburgh, PA, USA

#### Key ideas

- Much of the literature for managing patients admitted to the hospital with DKA and HHS focuses on acute management. However, goal-directed therapy of these hyperglycemic emergencies extends beyond acute management.
- Hypoglycemia and hypokalemia are commonly encountered complications of therapy that can adversely affect patient morbidity and mortality in the hospital. Ineffective transitioning from intravenous to subcutaneous insulin results in recurrent hyperglycemia and DKA.
- Patients presenting with hyperglycemia emergencies are at risk for adverse outcomes beyond the acute hospitalization, including frequent hospital readmissions, and mortality. This indicates a need for addressing issues that extend beyond acute management. Protocols targeting metabolic and glycemic management pitfalls are effective at reducing the frequency of adverse events, but there is a need to identify patients at risk for recurrent hospital admissions with DKA and HHS.

#### Summary

The most commonly identified risk factors for hyperglycemic crisis include insulin omission and infection. Alcohol use, pancreatitis, and new onset diabetes are also frequent causes.^20^ Insulin omission can occur due to financial constraints, mental stress, fear of hypoglycemia, a misunderstanding about stopping insulin when ill, or a maladaptive desire to lose weight. While acute management is often protocolized, several pitfalls can be encountered during subsequent care. These include stopping the insulin infusion prior to anion gap closure, failure to overlap subcutaneous and intravenous insulin, and recurrent hyperglycemia and increased anion gap after transition. Hypoglycemia and hypokalemia are common (occurring in 12%-35%^21^ and 16%-62%^22^ of patients, respectively) and associated with an increase in hospital mortality. Patients are also at risk for hospital readmission and have an increase in mortality at one year. Potentially modifiable risk factors for readmission include poor glycemic control, comorbid depression, substance use and psychiatric illness, underinsurance, and homelessness.

### Diabetes in the Emergency Room

Justin Yan, BSc (Hon), MD, MSc, FRCPC

Western University, London, ON, Canada

#### Key ideas

- Critical hypoglycemia is common and usually presents as acute neurologic manifestations in the ED.
- Psychosocial factors are important to consider when patients with DKA present to the ED, particularly in the younger adult age group.
- Although less common, HHS patients are generally older, have type 2 diabetes (T2D), and are much sicker than patients with DKA.

#### Summary

Hypoglycemia precipitating ED admission can occur due to chronic tight glycemic control, missed meals, increased energy output, or too much insulin or hypoglycemic medication. Patients often have neurologic symptoms, including confusion, seizures, and coma. Treatment includes oral or intravenous dextrose, glucagon, or octreotide for a sulfonylurea overdose. Providers should be cognizant of hypoglycemia unawareness and the Somogyi phenomenon contributing to presentation. Diabetic ketoacidosis occurs most often in patients with type 1 diabetes, and the mortality is approximately 1%. Psychosocial factors often play a role, in particular in younger patients. Providers should be aware of “euglycemic DKA” occurring in some populations. Hyperglycemic hyperosmolar state occurs more often in patients with T2D, the elderly, and those with comorbidities. Patients have a higher mortality rate than in DKA, a longer prodromal illness, a larger fluid deficit, and more commonly present with comorbidities. Management includes aggressive fluid repletion and treatment of comorbid illnesses.

## Session 3: Computer-Guided Insulin Dosing • Sponsored by Glytec

Moderator: Amisha Wallia, MD, MS

Northwestern University Feinberg School of Medicine Chicago, IL, USA

### Evaluating a Health System’s Glycemic Health and Next Steps

Damon Tanton, MD

AdventHealth Diabetes Institute, Orlando, FL, USA

#### Key ideas

- Inpatient insulin management has historically been associated with poor glycemic outcomes, as systems have relied on reactive, sliding scale therapies, and have underleveraged technology and automation.
- Centers for Medicare & Medicaid Services (CMS) will likely require comprehensive tracking and reporting of glucometrics, as we continue to transition from a fee-for-service (volume-based) reimbursement paradigm to one that is anchored around value-based care.
- In anticipation of this requirement, and in effort to reduce cost and optimize glycemic care, AdventHealth conducted a system-wide analysis of the impact of suboptimal inpatient glycemic management on seven of its Central Florida Hospitals.

#### Summary

As 50% of all medication errors involve insulin, it is critical that its administration be standardized and, when continuous, automated via computerized protocols. As CMS has developed a severe hypoglycemia measure to address the issues with glycemic mismanagement, it is also essential that hospitals develop a comprehensive approach and an effective system for self-monitoring. An exhaustive analysis of 43 659 insulin-requiring patients at seven AdventHealth hospitals over 12 months revealed that, compared to patients with controlled blood sugar, those with severe hypoglycemia had a cost $10 405 more per inpatient stay ($7.7 million, total), a length of stay of 6.6 more days, a 61.5% higher readmission rate, and a threefold higher mortality rate. These findings led to the clinical implementation of several new strategies, to include personalized insulin management using an eGlycemic Management System (eGMS), defaults to basal-bolus regimens, implementation of electronic surveillance, and more aggressive training of staff around best practices.

### Reducing Critical Hypoglycemia Through Quality Improvement Initiatives and Implementation of an eGlycemic Management System

Debra Dudley, BS, CDE, RN

AdventHealth Waterman, Tavares, FL, USA

#### Key ideas

- AdventHealth Waterman Hospital saw an opportunity to improve patient safety by reducing critical hypoglycemic events (blood glucose [BG] <40), with the implementation of quality improvement initiatives and the utilization of eGMS.
- Four causes of preventable hypoglycemic events were identified and targeted for improvement: stacking insulin, failure to follow policy, failure to choose a correct initial insulin dose, and failure to adjust insulin with a change in patient needs.
- After the implementation of eGMS, preventable hypoglycemic events were reduced by 62.6%.

#### Summary

AdventHealth Waterman is a “typical” 269-bed hospital, situated in the slow-paced, lake-front town of Tavares, Florida (population 16 000, average age of 67, and 14% incidence of diabetes). As its characteristics are generalizable to many hospitals across the country, Waterman has often been utilized to pilot studies for the entire AdventHealth System. One of such initiative was the creation and development of a comprehensive, interdisciplinary glycemic management team, which leveraged a new eGMS and robust informatics to reduce clinical errors and improve patient safety. In addition to significantly reducing rates of severe hypoglycemia and costs ($350 000 over two years), these efforts also led to greater adherence to hospital policies, improved rates of testing for all insulin users, easier hospital-to-home transitions for patients using insulin, and a 75% reduction in new phone calls to clinicians that were related to glycemic management.

### Utilization of Computer-Guided Insulin Dosing Decreases Hypoglycemia Adverse Drug Events, Length of Stay, and Cost of Care at a Large Pacific Northwest Health System

Thérèse Franco, MD, SFHM

CHI Franciscan, Tacoma, WA, USA

#### Key ideas

- Effective technology does not obviate the need for a human connection; it brings people together with easy access to the data they need for shared decision making on a personalized plan of care for the patient in front of them.
- Automation of daily dose adjustments allows providers to focus on the big picture.
- Continuous and predictive data analysis allows for more personalized care in the dynamic hospital environment.

#### Summary

Catholic Health Initiatives Franciscan (CHIF), located in South Puget Sound, Washington, is a system comprising eight acute care hospitals (802 beds, in aggregate) with a staff of approximately 4200 providers, 4000 nurses, and 150 pharmacists. In 2016, in response to higher rates of hypoglycemia and amid cultural and workflow barriers, CHIF leadership created a glycemic steering committee, which spearheaded an overarching glycemic management strategy. This initiative, which was anchored to a new eGMS, coordinated care by the way of order set revision, clinician education, pharmacist-led patient identification, glucometrics, and monthly process reviews. In addition to resulting in a downward trajectory for direct cost of care, these efforts led to substantial reductions in the rate of hypoglycemic events (44%, *P* < .001) and length of stays for both diabetic ketoacidosis and hyperglycemic hyperosmolar state patients (1.4 days, *P* < .001) and those with a diagnosis of diabetes (0.5 days, *P* < .033).

## Session 4: Coronavirus Disease 2019 and Diabetes

Moderator: Guillermo Umpierrez, MD, CDE

Emory University, Atlanta, GA, USA

### Panel Speakers

Shivani Agarwal, MD, MPH

Albert Einstein College of Medicine, The Bronx, New York, USA

Carlos E. Mendez, MD, FACP

Medical College of Wisconsin, Milwaukee, WI, USA

Robert Rushakoff, MD

UCSF, San Francisco, CA, USA

Jane Jeffrie Seley, DNP, MPH, MSN, GNP, RN, BC-ADM, CDCES, CDTC, FADCES, FAAN

Weill Cornell Medicine, New York, NY, USA

Guillermo Umpierrez, MD, CDE

Emory University, Atlanta, GA, USA

#### Key ideas

- Those with diabetes and/or hyperglycemia are at increased risk for worsening complications of COVID-19 and have increased morbidity and mortality.
- Presentations of patients with severe hyperglycemia, diabetic ketoacidosis (DKA), and in some cases, severe insulin resistance (COVID and non-COVID) appear to be clinically different phenotypes and presentations than previously seen; there have also been reported changes in insulin sensitivity with infection.
- Implementing novel methods of delivery of care to decrease personal protective equipment, bundle care, and decrease face-to-face time, such as continuous glucose monitoring (CGM), is needed but may be complex to implement depending on institution resources. The Food and Drug Administration (FDA) has noted “enforcement discretion” on hospital use of CGM.
- Additional protocols that save staff time, exposure, and other resources have been planned and implemented at many hospitals at a record pace. One successful example is the use of a subcutaneous insulin protocol for mild to moderate DKA, based on the previously published work.^19^
- The FDA released a new guidance “FAQs on Home-Use Blood Glucose Meters Utilized Within Hospitals During the COVID-19 Pandemic.” This allows patients to temporarily assist in monitoring their own blood glucose (BG) during hospitalization.

#### Summary

While institutional and statewide experiences with COVID-19 may differ considerably, much can still be learned by comparing healthcare delivery strategies and discussing clinical cases. One must first understand if those with diabetes mellitus are at higher risk for contracting COVID-19 disease, what the risk COVID-19 poses for those with DM, and how that risk is mediated by glucose control. At the time of the presentation, there were no data to support that there was an increased risk of having diabetes and being at risk for getting infected with SARS-CoV2. However, there were data from China, Italy, and the United States that those with diabetes have increased morbidity and mortality once infected.^19,23,24^ The interplay between obesity, diabetes, and glycemic control and outcomes is not clear; however, there is evidence that those with obesity and separately those with diabetes do worse clinically from COVID-19 disease. Poor glycemic control does appear to be detrimental to COVID-19-related morbidity and mortality.^25^

In addition, there are clearly pathological differences in the presentations of severe hyperglycemia, DKA, and insulin resistance that are being identified and treated in the setting of the COVID-19 pandemic. These have been described in other countries, but also across the United States. These case presentations are clinically divergent from pre-COVID-19 era case descriptions and include DKA in those with pre-DM, type 2 diabetes, or no history of diabetes, and in other cases, severe insulin resistance requiring extremely high amounts of insulin. However, interestingly, some patients with previous diabetes are presenting with COVID-19 and have de-escalating diabetes medication needs, specifically much less insulin than would be typically expected. Much is still unknown about this virus and the interplay between the virus, islet cell biology, insulin sensitivity, and insulin resistance; it will be critically important to share cases, management strategies, and data between institutions so that we can learn from one another. We will also need to pay critical attention to those patients who are in minority groups and/or in socio economically disadvantaged groups.

Novel care management strategies, such as telemedicine, telehealth, and technology, have and will need to be continually adopted. Several of these strategies have been initially intended to decrease the use of PPE, and decrease potential spread of the virus to those caring for patients and to other patients in the hospital. In New York and other places, some institutions have been studying the use of CGM to reduce point of care testing and the need for face-to-face interactions with these patients. These types of strategies need to be studied, given the fact that the FDA has noted “enforcement discretion” only, and there does appear to be wide variability in the ability to implement these across institution types. Additional protocols that save staff time, exposure, and other resources have been planned and implemented at many hospitals at a record pace. One successful example is the use of a subcutaneous insulin protocol for mild to moderate DKA, based on the previously published work.^19^

The FDA also released a new guidance “FAQs on Home-Use Blood Glucose Meters Utilized Within Hospitals During the COVID-19 Pandemic.”^26^ This allows patients to temporarily use their home BG meter during hospitalizations or allows hospitals to dispense a home meter to them, so that patients can then assist in monitoring their own BGs during hospitalizations. This plan could be utilized at a time of nursing, staff, or lab constraints during a surge.

Everyone can agree that the advent of COVID-19 has changed diabetes healthcare delivery in the hospital, and we need to continue to come together to combat this virus and make more of the unknown, known.

## Session 5: Technology

Moderator: Robert Rushakoff, MD

UCSF, San Francisco, CA, USA

### Pumps, Closed Loop, and Do-It-Yourself

Andjela Drincic, MD, FACP

University of Nebraska Medical Center, Omaha, NE, USA

#### Key ideas

- Safe insulin pump use in the hospital requires proper patient selection, policy-driven process, and effective patient-staff communication.
- Successful perioperative use of continuous subcutaneous insulin infusion (CSII) requires a defined decision-making process determining if CSII can be used intraoperatively and clearly outlined care process models that include provisions for transitions of care.
- Closed-loop systems and do-it-yourself (DIY) systems, while appealing, are best used in manual mode because of the current limitations of technology and hospital systems.

#### Summary

Over five million patients with type 1 and type 2 diabetes use insulin pumps and their presence in the hospital is becoming more frequent.^27,28^ Insulin pumps, compared to basal bolus insulin, can be used safely in the hospital with similar blood glucose (BG) outcomes and greater patient satisfaction. Consensus from multiple societies, including American Diabetes Association, American Association of Clinical Endocrinologists, Endocrine Society, and Diabetes Technology Society (DTS), support the use of insulin pumps in the hospital. The organizations recommend institutional policy and procedures for CSII use, pump order sets, a signed agreement with the patient, personnel familiar with CSII therapy, and criteria for proper patient selection. Insulin pumps can also be used safely in the perioperative setting if defined decision-making processes are used and clearly outlined care process models (including provisions for transitions of care) are in place.^29^ Closed-loop and DIY systems are also becoming more common in the inpatient setting, but are best used in manual mode in the hospital because of the current limitations of technology in the inpatient setting.

### Continuous Glucose Monitoring Today

Guillermo Umpierrez, MD, CDE

Emory University, Atlanta, GA, USA

#### Key ideas

- Large, multicenter studies on the accuracy of continuous glucose monitoring (CGM) are needed for Food and Drug Administration (FDA) clearance to use CGM in the hospital setting.
- Education and training programs for hospital personnel are needed to develop simplified systems for data transmission from the bedside to the nursing station (continuous glucose profile).
- Accurate CGM systems combined with automatic insulin dosing systems (closed loop) will facilitate glycemic control and reduction of hypoglycemia and hyperglycemia in patients with diabetes.

#### Summary

There are several available CGM systems including intravascular (venous and arterial), subcutaneous, and transdermal. Continuous glucose monitoring can provide benefit over intermittent point of care BG testing in the hospital to prevent severe hypo- or hyperglycemia. The mean absolute relative difference (MARD) varies widely among studies/devices in the intensive care unit (ICU), but more recent data demonstrate improving reliability of these devices. However, limitations do still exist with some devices in the ICU setting including (1) measurement lag time; (2) substance interference; (3) lack of evidence of accuracy during periods of arterial hypotension, hypothermia, or hypoxia; and (4) thrombus/infectious complications of intravascular devices. In the non-ICU setting, the MARD is improving, especially in the hyperglycemia range, but limitations remain in the hypoglycemia range as well as with substance interference, real-time transmission to nursing stations, new technology to hospital personnel, the risk of information overload, and cost.^29^ While new technology is promising, future studies are needed for FDA approval. Education and training programs for hospital personnel are needed as well as simplified systems for data transmission.

### Continuous Glucose Monitoring in the Pipeline

Jeffery Joseph, DO

Thomas Jefferson University, Philadelphia, PA, USA

#### Key ideas

- Vascular hospital CGM systems sample blood intermittently from a catheter placed within a radial artery, a peripheral vein, or the superior vena cava and then into an external flow-through glucose sensor; the GlucoScout and Optiscanner are FDA approved and available for clinical use in the United States.
- Commercial subcutaneous CGM systems are being used in clinical trials in the hospital evaluating the use of real-time CGM data with alerts and alarms at the bedside to avoid hypoglycemia, and the use of nursing protocols that calculate prandial and correction doses of subcutaneous insulin based upon the CGM system’s trend data (glucose concentration, direction of change, and rate of change).
- Continuous glucose monitoring systems in combination with validated nursing protocols for insulin dosing have great potential to improve overall BG control (increased percentage of time in the desired target glucose range), while minimizing the incidence and degree of hyperglycemia, hypoglycemia, and glycemic variability.

#### Summary

Several vascular hospital CGM systems that sample blood intermittently from a catheter placed within a radial artery, a peripheral vein, or the superior vena cava and then into an external flow-through glucose sensor are FDA cleared and available for use in the United States including the GlucoScout and Optiscanner. Limitations include increased risk for intravascular thrombus formation, premature catheter failure, and sampling error from dextrose infusions or dilution due to residual flush solution. Closed-loop artificial pancreas systems are able to “clamp” the glucose concentration in a desired range by automatically titrating intravenous infusions of insulin and dextrose. The current clinical trials using commercial subcutaneous CGM systems are evaluating the use of real-time CGM data with alerts and alarms at the bedside to avoid hypoglycemia, and nursing protocols that calculate prandial and correction doses of subcutaneous insulin based upon the CGM system’s trend data (glucose concentration, direction of change, and rate of change) in the non-intensive care unit setting.

### A Virtual Glucose Management System

Robert Rushakoff, MD

UCSF, San Francisco, CA, USA

#### Key ideas

- Communication within and between teams is crucial for effective inpatient glucose management.
- Virtual Glucose Management System (vGMS) is allowed for a significant infrastructure, trained staff, and succinct notes.
- Next steps in vGMS are to expand vGMS to local affiliated hospitals, apply artificial intelligence to vGMS, and learn from patients and experience to make fundamental changes in the inpatient glucose management system.

#### Summary

Despite over two decades of extensive work creating infrastructure such as order sets, work flows, and education on inpatient diabetes management with improved glucometrics and low rates of hypoglycemia, audits continued to show inappropriate initial insulin orders and clinical inertia for both attending staff and house staff at UCSF. With the implementation of electronic health record and creation of daily reports identifying patients in real time who were out of control, the vGMS was established and could provide clinicians with appropriate glucose management recommendations in a timely manner via a brief note in the chart and just-in-time education.^30^ Through these efforts, there was a significant decrease in hospital-wide hyperglycemia and hypoglycemia.^31^ The next steps are to expand vGMS to local affiliated hospitals, apply artificial intelligence to vGMS, and learn from patients and experience to make fundamental changes in the inpatient glucose management system.

### Computer-Guided Insulin Dosing

Kristen Kulasa, MD

UCSD, La Jolla, CA, USA

#### Key ideas

- Computer-guided intravenous (IV) insulin infusion has higher staff satisfaction, better compliance with protocol, more time with glucose in range, and less variability than typical paper protocols.
- UC San Diego Health has been using a “home-grown” computerized insulin infusion protocol since 2004 and successfully integrated the algorithm directly into the EPIC medication administration record (MAR) in 2017 with safety and efficacy data comparable to published and commercial products.
- Computer-guided subcutaneous insulin dosing is also available using standardized order sets with built-in clinical decision support, commercial products with full basal, bolus insulin dosing, or “home-grown” options such as UC San Francisco’s subcutaneous insulin calculator for continuous nutrition.

#### Summary

Computer-guided IV insulin infusion has higher staff satisfaction, better compliance with protocol, more time with glucose in range, and less variability than typical paper protocols. UC San Diego Health has been using a “home-grown” computerized insulin infusion protocol since 2004 and successfully integrated the algorithm directly into the EPIC MAR in 2017 with safety and efficacy data comparable to the published commercial products. This computerized insulin infusion calculator is used in a variety of hospital and clinical settings, including ICU, non-ICU, emergency department, hyperglycemic emergencies, diabetes in pregnancy, and patients with renal failure. Computer-guided subcutaneous insulin dosing is also available using standardized order sets with built-in clinical decision support, commercial products with full basal, bolus insulin dosing, or “home-grown” options such as UC San Francisco’s subcutaneous insulin calculator for continuous nutrition. Leveraging technology to improve adherence to protocols, provide clinical decision support, and assist with both IV and subcutaneous insulin dosing is possible and is becoming increasingly more common.

## Session 6: Hypoglycemia

Moderator: Jane Jeffrie Seley, DNP, MPH, MSN, GNP, RN, BC-ADM, CDCES, CDTC, FADCES, FAAN

Weill Cornell Medicine, New York, NY, USA

### Prediction Models for Inpatient Hypoglycemia

Nestoras Mathioudakis, MD, MHS

Johns Hopkins University, Baltimore, MD, USA

#### Key ideas

- Hypoglycemic agents account for over half of adverse drug events in hospitalized patients. Iatrogenic hypoglycemia in the hospital has been associated with cardiac and neurologic symptoms, increased costs, longer length of stay, increased nursing resources, and mortality.
- Real-time alerting of patients at high risk of near-term iatrogenic hypoglycemia has the potential to reduce rates of this potentially serious adverse event. Early prediction models of iatrogenic hypoglycemia in the hospital setting had poor predictive accuracy owing to the small sample sizes and limited number of clinical predictors. Machine learning methods using very large electronic medical record (EMR) datasets can substantially improve the predictive accuracy of iatrogenic hypoglycemia models. Recent machine learning prediction models, while highly accurate, have used a prediction horizon of the entire admission, which limits the ability to translate such models into real-time informatics alerting to modify prescribing practices of hypoglycemic medications throughout the course of a patient’s hospitalization. Prediction models using a shorter prediction horizon (e.g. next 24 hours) would offer greater value to inpatient clinicians considering that antihyperglycemic medications are typically adjusted on a daily basis.
- Machine learning prediction models integrated into the EMR for reducing the incidence of iatrogenic hypoglycemia should be validated both internally and externally to assess their generalizability across different patient populations and health systems.

#### Summary

A significant proportion of inpatients have diabetes or hypoglycemia and almost two million hospital stays annually in the US are affected by adverse drug events caused by hypoglycemic agents. Iatrogenic hospital hypoglycemia increases the risk of poor clinical outcomes as well as costs and resources needed. An iatrogenic hypoglycemia prediction model (using machine learning) integrated with a patient’s EMR could provide real-time alerts to healthcare professionals. Using a shorter prediction horizon would also help with providing useful real-time predictive data. Timely notification would allow healthcare professionals to predict or treat iatrogenic hypoglycemia at an early stage. Such notifications could inform a clinical strategy based on a patient’s current medications and preferences. Validating a prediction model using both internal and external EMR data sets is necessary to assess generalizability of the model and to allow for implementation of the model in multiple hospitals.

### Implementation of a Hypoglycemia Prevention Program

Nadine E. Palermo, DO

Brigham and Women’s Hospital/Harvard Medical School, Boston, MA, USA

#### Key ideas

- Hypoglycemia in the hospital setting is associated with poor outcomes, including longer length of stay and increased risk of death.
- There are several factors that have been associated with the increased risk of hypoglycemia, including older age, impairment in renal function, change in nutritional intake, and interruption in nutrition and/or glucose monitoring.
- Electronic health record (EHR)-based tools including real-time glucometrics can be helpful in the development of a hypoglycemia prevention program.

#### Summary

Hypoglycemia in hospitalized patients with diabetes is often associated with risk factors resulting in prolonged action of antidiabetic drugs or decreased caloric intake.^32^ Because hypoglycemia can lead to increased length of the patient’s hospital stay and increased risk of death, education of the healthcare staff is important to help reduce the incidence of hypoglycemia in the hospital. The development of a hypoglycemia prevention program can be potentially aided by real-time glucometrics programs and use of other EHRs, which can rapidly provide real-time readings of blood glucose levels and allow hospitals to see trends and compare their performance against other hospitals.

### Improving Insulin Safety in the Hospital Setting: Errors Be Gone

Jane Jeffrie Seley, DNP, MPH, MSN, GNP, RN, BC-ADM, CDCES, CDTC, FADCES, FAAN

Weill Cornell Medicine, New York, NY, USA

#### Key ideas

- The Emergency Care Research Institute (ECRI) and the Institute for Safe Medication Practices (ISMP) have combined efforts to form a nonprofit organization that promotes patient safety by highlighting adverse effects, near misses, and unsafe conditions in various healthcare settings. ISMP became an affiliate of ECRI in 2019. ISMP plays a key role in reducing insulin errors by advocating for improvements in drug labeling, packaging, preparation, and administration.
- Electronic solutions can be built into the EHR to remind prescribers to order basal insulin or an acceptable alternative for patients with type 1 diabetes (T1D) throughout the hospital stay. Missed doses stem from a number of prescriber, nurse, and pharmacy errors that could be reduced via a combination of EHR reminders and diabetes education.
- Structured insulin order sets with computerized provider order entry in the EHR can improve insulin prescribing patterns and lower rates of hypo- and hyperglycemia. Order sets should have separate orders for basal, prandial, and correctional insulin so that the nurse is able to hold the prandial insulin if the patient is not eating, but still administer insulin to correct any hyperglycemia.

#### Summary

Common sources of insulin errors in the hospital setting are dose omission, overdosage, and underdosage.^33^ These errors can lead to dysglycemia and poor outcomes. The ECRI and its affiliate, ISMP, are working together to educate healthcare professionals on unsafe conditions in the hospital and possible strategies for prevention; they advocate for improvements in drug labeling, packaging, and processing because many insulin errors result from drug packaging and names that look similar. Creating alerts in the EHR that remind prescribers to order basal insulin for patients with T1D could lead to a decreased number of insulin errors since these errors often occur because of prescriber, nurse, or pharmacy errors. Healthcare professionals should also be informed of the value of comprehensive weight- and sensitivity-based insulin order sets to promote sensible insulin dosing.

## Session 7: Data and Cybersecurity

Moderator: Jane Jeffrie Seley, DNP, MPH, MSN, GNP, RN, BC-ADM, CDCES, CDTC, FADCES, FAAN

Weill Cornell Medicine, New York, NY, USA

### Managing Glucose Data in the Clinical Laboratory

Martha Lyon, PhD, DABCC, FAACC

University of Saskatchewan, Saskatoon, Canada

#### Key idea

- The clinical accuracy of glucose meters has traditionally been assessed using error grids like the Clarke Error Grid, the Parkes Error Grids, and the Surveillance Error Grid.
- The insulin dose error assessment (IDEA) grid is a clinical accuracy tool that describes the difference in glucose measurement in terms of insulin dosing error categories.
- Information obtained from the IDEA grid complements glucose meter performance interpretation provided by the Parkes Error Grids and the Surveillance Error Grid.

#### Summary

Methods that measure glucose need to have increased clinical accuracy, which is defined as making clinical treatment decisions based on test results which could affect clinical outcome. Over the years, error grids were established to assess clinical decision-making based on the difference between the results obtained by two analytical methods. The IDEA is a novel clinical tool that can compare different glucose methods. This tool depicts the frequency and extent of insulin dose error attributed to glucose method errors. Insulin dose error assessment grid is fully customizable and can be utilized for institutional specific insulin dose protocols, enabling assessment of clinical risk and the evaluating errors in insulin administration.

### Cybersecurity of Wearable Diabetes Devices and Hospital Diabetes Devices

David C. Klonoff, MD, FACP, FRCP (Edin)

Mills-Peninsula Medical Center, San Mateo, CA, USA

#### Key ideas

- Diabetes device cybersecurity assures that data and command information will be accurately transmitted between a wireless device and a monitor or controller.
- The current coronavirus disease 2019 pandemic has increased the cybersecurity vulnerability of hospitals that have incorporated temporary medical facilities or increased their use of telemedicine.
- Diabetes devices with wireless communication that require sound cybersecurity can include pumps, smart pens, blood glucose monitors, continuous glucose monitors, and closed-loop systems.

#### Summary

Cybersecurity is the protection of connected medical devices from unauthorized disclosure, modification, and loss of function. Sound cybersecurity protects confidentiality integrity and availability of data. Cybersecurity is one feature of a connected medical device, in addition to safety, effectiveness, privacy, usability, and cost. Wearable and hospital devices contain personal, medical, and financial information about patients that can be targets for hackers. No patient harm has been reported through a cybersecurity breach of a diabetes device. The Food and Drug Administration (FDA) regulates device cybersecurity and requires demonstration of robust cybersecurity for wireless device clearance. The FDA does not test devices for cybersecurity. Therefore, meeting standards for cybersecurity, like the Diabetes Technology Society (DTS) Cybersecurity Standard for Connected Diabetes Devices, provides a path for a manufacturer to provide assurance to patients and healthcare professionals about the safety of wireless diabetes devices. Hospitals are vulnerable to security breaches because they often use outdated operating systems in an unpartitioned network and they may lack the resources to address cyber threats.

### Liability for Software in the Hospital

Tonya Newman, JD

Neal, Gerber & Eisenberg LLP, Chicago, IL, USA

#### Key ideas

- To the extent feasible, expanding the indications for CGM into appropriate inpatient settings has been advocated.
- The question of whether to seek FDA approval, whether to share any Software as a Medical Device (SaMD), and the associated potential exposures should be carefully considered and discussed with the hospital’s counsel and risk management team, to ensure that the hospital’s potential exposures are minimized to the greatest extent possible.
- If hospitals are going to develop their own software systems to store and summarize the data from continuous glucose monitors, then consider collaborative working groups to ensure that all potential exposures are being considered and appropriate expertise is available to mitigate those risks

#### Summary

The FDA likely would consider an insulin dosing system to be an SaMD, and any hospital developing and sharing such a system probably should first seek FDA approval and otherwise comply with the applicable regulatory requirements. Similarly, software functions using an attachment to the mobile platform to measure glucose levels are subject to regulatory oversight rather than discretion. Failure to obtain FDA approval could (and likely would) also be used against a hospital defendant in malpractice and other litigation. Developing and sharing SaMD should proceed only after the associated potential exposures have been carefully considered and discussed with the hospital’s counsel and risk management team, to ensure that the hospital’s potential exposures are minimized to the greatest extent possible.

## Session 8: Special Situations

Moderator: Amisha Wallia, MD, MS

Northwestern University Feinberg School of Medicine, Chicago, IL, USA

### Managing Steroid-Induced Hyperglycemia

Felicia A. Mendelsohn Curanaj, MD

Weill Cornell Medicine, New York, NY, USA

#### Key ideas

- Glucocorticoids (GCs) cause hyperglycemia through multiple mechanisms, including a decrease in pancreatic insulin secretion, an increase in hepatic gluconeogenesis, a decrease in insulin sensitivity, and an impairment of the incretin effect.
- The hyperglycemic effect is influenced by GC potency, duration of action, route of administration, and duration of use.
- Factors to consider when managing steroid-induced hyperglycemia include history of type 1 or type 2 diabetes, eating status, and steroid factors, such as the type of GC, dose, and dosing schedule.

#### Summary

Hyperglycemia can often occur in patients receiving GCs, regardless of whether or not they have a history of diabetes. Glucocorticoids can cause hyperglycemia through a decrease in pancreatic insulin secretion, an increase in hepatic gluconeogenesis, a decrease in insulin sensitivity, or an impairment of the incretin effect. The correlation between GC usage with the degree of blood glucose elevation is unclear, because patients have different diabetes histories and often receive different GC formulations, doses, and dosing schedules, leading to varying hyperglycemic effects. All of these factors must be considered when managing steroid-induced hyperglycemia. Glucose monitoring is recommended for patients on GCs, especially those with diabetes or a history of diabetes. NPH (Neutral Protamine Hagedorn) insulin, basal-bolus insulin, or modified basal-bolus insulin therapy can be used to manage hyperglycemia in patients with preexisting diabetes.^34^ There are limited data on the effects of oral agents and non-insulin injectables on steroid-induced hyperglycemia.

### Managing Hyperglycemia During Inpatient Nutritional Support

Andjela Drincic, MD, FACP

University of Nebraska Medical Center, Omaha, NE, USA

#### Key ideas

- Hyperglycemia during enteral (EN) and parenteral (PN) nutrition can be prevented/ameliorated by
  - use of diabetes specific formulae in EN and
  - decreasing the dextrose content to 150 to 200 g/day in PN.

- Basal bolus principles of therapy where nutritional coverage can be calculated based on carbohydrate load can be applied in EN and PN therapy:
  - in EN, the basal bolus ratio should be 40/60 to 50/50 (careful not to overbasal);
  - in PN, adding regular insulin in a PN bag according to insulin dextrose ratio is appropriate.

- Prevention of hypoglycemia with proactive order sets is crucial to ensure safety in case of nutrition interruption.

#### Summary

A significant percentage of patients receiving nutritional support experience hyperglycemia, with increased prevalence in patients receiving PN nutrition in comparison with patients receiving EN nutrition.^35^ Hyperglycemia can be prevented using lower concentrations of dextrose in PN and using a diabetes-specific formula in EN, which has been shown to improve mortality in patients with type 2 diabetes in the intensive care unit. Further issues may arise in treating hyperglycemia with EN if the tube feed is discontinued or if the rate is changed; proactive order sets must be prepared to ensure patient safety if this happens or if PN is interrupted. Future studies using continuous glucose monitoring should be conducted with the hope of creating accurate insulin-dosage calculators for widespread usage.

### Transplant Management

Amisha Wallia, MD, MS

Northwestern University Feinberg School of Medicine, Chicago, IL, USA

#### Key ideas

- Randomized control trial data in different transplant groups have been mixed, but it is likely that strict to moderate control (110-140 and 140-180 mg/dL) is better for transplant-related outcomes than poor glycemic control (>180 mg/dL).
- Insulin has been studied in the transplant population, can be dose adjusted for renal dysfunction, and has few drug-drug interactions.
- Other classes of oral and injectable medications have to be evaluated on a case-by case basis, as dose adjustment for renal function, side effect profiles, drug-drug interactions, and potential comorbidities need to be taken into account in this specialized population.

#### Summary

After receiving a transplant, patients who experience hyperglycemia can be at an increased risk for transplant rejection because of increased inflammation and endothelial dysfunction.^36,37^ Many medications, such as corticosteroids and calcineurin inhibitors, used for immunosuppression to reduce risk of transplant rejection and induce hyperglycemia through multiple mechanisms, increasing the probability of poor clinical outcome. Using insulin may alleviate symptoms associated with hyperglycemia in the short term because of its regulatory effect on inflammation and immunity. The dose of insulin can be decreased as estimated glomerular filtration rate approaches 45 mL/ min to maintain glycemic control and reduce the risk of hypoglycemia. Further studies on noninsulin treatment options such as oral or injectable medications need to be done to compare the effectiveness in both short-term and long-term outcomes and to assess potential comorbidities and side effects. Coronavirus disease 2019 patients with diabetes or hyperglycemia were characterized with recurrent uncontrolled hyperglycemia and an increased mortality rate.^38^

### Bariatric Surgery

Erik Dutson, MD, FACS

UCLA, Los Angeles, CA, USA

#### Key ideas

- There is now sufficient clinical and mechanistic evidence to support the inclusion of metabolic surgery among the antidiabetes interventions for people with type 2 diabetes (T2D) and obesity.
- For morbidly obese patients with T2D, not having surgery is much more dangerous than having surgery in the long term.
- Bariatric surgeons and endocrinologists can and should work together to help these patients.

#### Summary

Obesity trends mirror diabetes trends in U.S. adults and obesity has surpassed tobacco use as the number one cause of preventable death. Obesity is a major risk factor for developing T2D, and the probability of attaining a normal body weight for an obese person is very low with diet and exercise alone.^39^ Over 90% of patients with T2D are overweight. Bariatric surgery is rapidly emerging as an antidiabetes intervention for T2D and obesity because of decreases in the severity of symptoms and low recurrence rates after the procedure. After treatment, the ghrelin signaling pathway is disrupted, decreasing the patient’s appetite and effectively helping the patient lose weight. Studies have shown that weight loss corresponds with better clinical outcomes and reduced recurrence rates after bariatric surgery. Future collaboration between bariatric surgeons and endocrinologists is necessary to ensure optimal patient care.

## Session 9: Glucometrics and Insulinometrics

Moderator: Elias K. Spanakis, MD

University of Maryland School of Medicine, Baltimore, MD, USA

### Glucometrics

Gregory Maynard, MD, MSc, MHM

UC Davis, Sacramento, CA, USA

#### Key ideas

- Glucometrics is the systematic analysis of blood glucose (BG) data, depicting glycemic control, glycemic exposure, hypoglycemia, and the interplay between them.
- Glucometrics complement outcomes and process measures for glycemic control programs, allowing for month-to-month tracking, comparative benchmarking, and active surveillance of inpatients.
- The CDC (Centers for Disease and Control and Prevention) has convened a panel to work toward public reporting to the National Healthcare Safety Network (NHSN) on hypoglycemia rates, using several variants of a National Quality Forum (NQF)-endorsed e-measure of hypoglycemia rates for benchmarking across hospitals.

#### Summary

Glucometrics is the systematic analysis of BG data, evaluating glycemic control, glycemic exposure, hypoglycemia, and the interplay between them. Glucometrics allows for real-time (active surveillance) as well as retrospective (month-to-month or biannual) tracking of inpatients. In this manner, it complements process measures for glycemic control programs, identifies opportunities for improvement, and can help improve outcomes. Currently, there is no consensus identifying the best metric for inpatient glycemic control. Institutions can define their own measures, keeping in mind clear definitions of unit analysis and attribution. There are also ongoing efforts to standardize glucometrics, facilitate reporting, and allow for comparative benchmarking. A reporting system developed by Society of Hospital Medicine allows for external benchmarking across more than 100 hospitals and across a variety of metrics. Those include patient-day or patient-stay weighted means, percent of patient days in range, percent of patient days with hypo- and hyperglycemia, and timeliness of hypoglycemia treatment. In addition, CDC has convened a panel to work toward national public reporting to the NHSN on hypoglycemia rates for benchmarking across hospitals. There are ongoing national efforts on behalf of the NQF as well as Centers for Medicare & Medicaid Services (CMS) to standardize glucometrics and performance goals.

### Insulinometrics

Curtiss B. Cook, MD

Mayo Clinic Arizona, Scottsdale, AZ, USA

#### Key ideas

- Insulinometrics is the systematic analysis and reporting of inpatient insulin therapy.
- Insulin is a high-risk medication important for the management of inpatient diabetes and should be tracked.
- Glucometrics and insulinometrics should be considered together.

#### Summary

Insulinometrics is the systematic analysis and reporting of inpatient insulin therapy. Insulin therapy is needed for most patients with diabetes and hyperglycemia in order to achieve recommended glucose goals. Yet the clinical inertia, defined as a failure to appropriately intensify insulin therapy, continues to be a barrier limiting the achievement of these goals. It is in part rooted in practitioners’ discomfort when prescribing insulin, which is recognized as a high-risk medication with a potential to cause significant harm when used in error. Therefore, analysis and understanding of insulin use and choice of therapy may guide institutions’ efforts to overcome clinical inertia. Identifying areas with a high rate of hypo- or hyperglycemia can trigger the analysis of how insulin is used. Given its high-risk classification, tracking insulin errors should be a top insulinometric. Insulin errors can be classified as wrong dose, wrong insulin, and whether or not the error reached the patient. Control charts can be developed to track details of insulin errors. Institutions can also track the number of insulin orders written and doses delivered. They can then analyze whether or how insulin therapy is being modified. Measuring patterns of insulin therapy against some type of glucometric may identify an opportunity to shift from correction-dosing to basal-bolus therapy. Glucometrics and insulinometrics should be utilized together to assess an institution’s management of glucose control and facilitate the achievement of goals.

## Session 10: Quality and Safety

Moderator: David Kerr, MD

Sansum Diabetes Research Institute, Santa Barbara, CA, USA

### Eat Your Medicine

David Kerr, MD

Sansum Diabetes Research Institute, Santa Barbara, CA, USA

#### Key ideas

- The inpatient administration of rapid-acting nutritional insulin may not match the timing of a meal, leading to dysglycemia.
- Smart insulin pens may have the potential to reduce in-hospital insulin errors.
- Insulin decision support tools may lead to improvements in glycemic control and financial benefits in the noncritical care setting.

#### Summary

There is a growing understanding of the interaction between food and medicine in the hospital setting. The inpatient administration of rapid-acting nutritional insulin may not match the timing of a meal, thus leading to hypoglycemia or hyperglycemia. Smart insulin pens are becoming more available and can log the timing and dose of insulin given. A recent study describing the use of smart insulin pens with memory and continuous glucose monitoring systems has shown that around 27% of boluses are late or missed completely. These pens may have the potential to reduce in-hospital insulin errors. Furthermore, layering decision support tools in the noncritical care hospital setting may also lead to improvements in glucose control, better clinical outcomes, and variations in the length of stay across hospitals and financial benefits. The incidences of diabetes and obesity continue to rise. Given the known association between diabetes and obesity and the risk of adverse events in hospitals, more attention should be given to optimizing lifestyle interventions, especially food choices before hospital admission to reduce individual risk.

### How to Design a Quality Improvement Project

Gregory Maynard, MD, MSc, MHM

UC Davis, Sacramento, CA, USA

#### Key ideas

- Quality improvement may improve health and reduce the cost of care.
- Following a roadmap for improvement, without skipping essential steps, may improve the chances of success of a quality improvement project.
- Plotting data over time may show progress visually and may help to avoid errors in interpreting common “before and after” analyses.

#### Summary

Quality improvement activities are essential to improving the health of the population, enhancing patient experiences and outcomes, and reducing the cost of care. However, about 80% of quality improvement projects fail to reach their stated objectives. Healthcare organizations can dramatically improve their chances of success by following a roadmap for improvement, without skipping essential steps. Defining the problem is the first and most important step. Process mapping, brainstorming, Pareto charts, and Fishbone diagrams may help identify key drivers of dysfunction in a system and thus generate interventions that address those key drivers. Plotting data over time in annotated run charts or statistical process control charts show progress visually and avoid errors in interpreting common “before and after” analyses. The COVID-19 crisis may decrease the funding of quality improvement hospital-based projects.

### Hospital Diabetes Management Programs

Roma Gianchandani, MD

University of Michigan, Ann Arbor, MI, USA

#### Key ideas

- Various specialized diabetes team models may be one of the best ways to manage diabetes in hospitalized patients.
- These initiatives may help reduce hospital readmissions and the length of stay of patients with diabetes.
- Hospital diabetes management programs may lead to better outcomes and financial savings for the hospital.

#### Summary

Different formats of diabetes management teams may include solo endocrine consultants, multidisciplinary and/or virtual consulting teams. Advanced practice providers are frequently utilized to take care of hospitalized patients with diabetes. Other members of the team may include residents, Endocrinology fellows, diabetes educators, nutritionists, pharmacists, case managers, and social workers. There is no single best program format, as every institution may have different needs and resources. An “automatic consult concept” may be useful in some of the medical units, which helps to avoid the delay of care of patients with diabetes. Specialized diabetes teams may help reduce hospital readmissions and the length of stay of patients with diabetes, especially if they are involved in diabetes care early in the admission. Hospital diabetes management programs may lead to better outcomes and financial savings for the hospital.

### Quality and the New Field of Endocrine Hospitalist

Mihail Zilbermint, MD, FACE

Johns Hopkins University School of Medicine, Baltimore, MD, USA

#### Key ideas

- Endocrine Hospitalist is the new field of endocrinology, modeled after the general hospitalist service.
- The initiative is designed to improve the quality of diabetes care in hospitalized patients.
- This model has proven its worth in reducing lengths of stay, hospital readmissions, and inpatient hypoglycemia.
- Inpatient diabetes telehealth approach may be reasonable for some hospitalized patients with COVID-19.

#### Summary

Endocrine Hospitalist is the new field of endocrinology, modeled after the general hospitalist service. It was designed to improve the quality of diabetes care in hospitalized patients. Because of its “make-whole” design, it requires an upfront financial investment from a hospital. The Hospital Readmissions Reduction Program is a Medicare value-based purchasing program that reduces payments to hospitals with excess readmissions. The readmission data for every hospital in the United States are available in the public domain and hospitals take pride in readmission reduction. The Endocrine Hospitalist model has proven its worth in reducing lengths of stay, hospital readmissions, and inpatient hypoglycemia, and providing a significant return on investment.^40,41^ Inpatient diabetes telehealth approach may be reasonable for some hospitalized patients with COVID-19 and dysglycemia.^42^ The Endocrine Hospitalist model should guide other community hospitals in their endocrine and glucose management initiatives.^43^

## Conclusion

The Hospital Diabetes Meeting’s sessions provided a comprehensive overview of different areas related to the inpatient management of diabetes. This event outlined the advances of diabetes technology in the hospital setting. Based on the rapid progress in this area, it is reasonable to anticipate increasing demand for the use of these technological approaches that will be dictated by hospital administrators, healthcare professionals, and/or patients with diabetes. This report provides important and novel information about how to improve the health care of hospitalized patients with diabetes without increasing the liability risk or compromising safety, privacy, or cybersecurity.
